# Functional relevance for associations between osteoporosis and genetic variants

**DOI:** 10.1371/journal.pone.0174808

**Published:** 2017-04-03

**Authors:** Kun Liu, Li-Jun Tan, Peng Wang, Xiang-Ding Chen, Li-Hua Zhu, Qin Zeng, Yuan Hu, Hong-Wen Deng

**Affiliations:** 1 Laboratory of Molecular and Statistical Genetics, College of Life Sciences, Hunan Normal University, Changsha, Hunan, China; 2 Physical Examination Department, Guangxi medical university First affiliated hospital, Nanning, Guangxi, China; 3 Center of Bioinformatics and Genomics, School of Public Health and Tropical Medicine, Tulane University, New Orleans, LA, United States of America; Yale University, UNITED STATES

## Abstract

Osteoporosis is characterized by increased bone loss and deterioration of bone microarchitecture, which will lead to reduced bone strength and increased risk of fragility fractures. Previous studies have identified many genetic loci associated with osteoporosis, but functional mechanisms underlying the associations have rarely been explored. In order to explore the potential molecular functional mechanisms underlying the associations for osteoporosis, we performed integrative analyses by using the publically available datasets and resources. We searched 128 identified osteoporosis associated SNPs (*P*<10^−6^), and 8 SNPs exert cis-regulation effects on 11 eQTL target genes. Among the 8 SNPs, 2 SNPs (*RPL31* rs2278729 and *LRP5* rs3736228) were confirmed to impact the expression of 3 genes (*RPL31*, *CPT1A* and *MTL5*) that were differentially expressed between human subjects of high BMD group and low BMD group. All of the functional evidence suggested the important functional mechanisms underlying the associations of the 2 SNPs (rs2278729 and rs3736228) and 3 genes (*RPL31*, *CPT1A* and *MTL5*) with osteoporosis. This study may provide novel insights into the functional mechanisms underlying the osteoporosis associated genetic variants, which will help us to comprehend the potential mechanisms underlying the genetic association for osteoporosis.

## Introduction

Osteoporosis is a skeletal disease characterized by low bone mineral density (BMD) and micro-architectural deterioration of bone tissue, leading to decreased bone strength and increased the risk of fracture[1]. Osteoporosis not only endangers the health and life quality of patients, but also brings huge economic burden to the global health. More than 2 million Americans suffered osteoporotic fractures in 2005 with treatment costs more than $17 billion [2]. Due to the aging of the population in the United States, it is expected that osteoporotic fractures rates will reach more than 3 million patients and $25.3 billion treatment costs over the next 25 years [3]. More and more developing countries will experience the rapid increase in the elderly population, which will lead to a greater number of individuals suffering from osteoporosis and fractures. It is expected that by 2050, up to half of the hip fracture patients will appear in Asia [4].

Genome-wide association studies (GWAS) have been successful at identifying a number of promising genetic variants that are associated with osteoporosis and related traits. However, the statistical association between OP and genetic variants were only established in the DNA level at the present stage, functional relevance has rarely been explored. Such established associations do not provide direct understanding into functions of significant candidate genes or regulation of genes expressions that functionally establish a connection between OP phenotype and gene code information.

Transcriptional regulation plays an important role in functional mechanisms underlying genetic association. The change of binding affinity between variants and regulatory factors altered by genetic variants may influence the transcription and/or translation of target proteins. Numerous public gene expression datasets are available, which are invaluable and may provide the functional data support for a better understanding of the association between variants and phenotype.

We combined the integrative analyses[5–8] (gene relationships among implicated loci, expression quantitative trait loci (eQTL) analysis, differential gene expression analysis and functional prediction analysis) results to research functional mechanisms for OP-associated genetic variants by utilizing the available data sources and analyzing available GWAS results. Performing the integrative analyses by using public data resources may strengthen our understandings in the molecular genetic mechanisms underlying complex diseases.

## Materials and methods

### Selection of OP-associated SNPs

Phenotype-Genotype Integrator (PheGenI) (www.ncbi.nlm.nih.gov/gap/PheGenI/) is a bioinformatics online tool that can provide robust view and download analystic data (including SNPs, genes and association results) for published studies[9]. Using phenotype “BMD” and “osteoporosis”, we identified 128 interesting SNPs (S1 Table) with *P* value <10^−6^ by searching PheGenI[10–15].

### Gene Relationship Across Implicated Loci (GRAIL)

Under normal circumstances, one disease-associated SNP has one or more predisposing genes or locus in the region near this SNP. In order to carry out the further functional studies, selecting genes located at the two sides of the associated SNP as potential candidate genes is a conventional method to identify the candidate genes[5]. With the purpose of discover more candidate genes, we performed GRAIL analysis (http://www.broadinstitute.org/mpg/grail/), which is an online statistical method that examining relationships automatically between seed regions and candidate genes were selected through PubMed abstracts to prioritizes the best candidate gene[16]. The seed regions are SNPs by searching PheGenI (250~500 kb flanking regions of the SNP). GRAIL analysis can select new potential candidate genes near OP-associated SNPs automatically[6].

### eQTL analysis

Variation occurs at the level of DNA may lead to the changes of genes expression, which subsequently account for a significant proportion of the phenotypic variance[6] (e.g. BMD) and susceptibility to OP. Therefore, eQTL analysis in specific tissues or cells is a favorable tool to identify candidate SNPs[17,18] and it is very important to study the functional molecular mechanism of the association by detecting whether the OP-associated SNP affects the levels of candidate gene expression. Some previous studies have found quite a lot of eQTLs in a variety of cells and tissues[19–23], we can search those databases quickly by eQTL Browser (http://eqtl.uchicago.edu/cgi-bin/gbrowse/eqtl/). We used this database to detect the eQTL effects of the identified OP-associated SNPs in monocytes and lymphoblastoid cell lines (LCLs). In humans vivo, the favorable working cell model for studying gene/protein expression patterns and their regulation mechanisms with reference to osteoporosis risk is monocyte[24,25]. Monocytes as the precursors of osteoclasts[26], related to osteoclast differentiation, activation, and apoptosis[27]. The expression level of genes are very similar in both human osteoblasts and LCLs and are enriched in pathways that are important in cellular growth and survival[28–31].

### Differential expression analysis

Three previous in vivo genome wide gene expression studies in our group using Human Genome U133 Plus 2.0 or U133A Arrays were performed to identify genes differentially expressed in monocytes or B cells between low and high BMD women., and we uploaded those data to GEO (gene expression omnibus) Datasets. The experimental procedures and data analysis were detailed in the original studies, and the GSE numbers are: GSE7158, GSE13850, GSE2208 [32–34]. The differential expression analysis (*P*<0.05) of for the identified eQTL genes between low and high BMD were detected by using *t*-tests.

### Functional prediction

For the identified eQTL SNPs, we used two online tools to predict the potential functions. F-SNP is a database to provide the latest information about already confirmed and presumed potential functional effect of SNPs at the multiple level, such as splicing, transcriptional, translational, and post-translational(http://compbio.cs.queensu.ca/F-SNP/). RegulomeDB[35] is an online public data resource, by integrating multiple data resources (such as high-throughput and experimental data) to perform predictions and annotations and then to identify potential functional variants (http://regulome.stanford.edu/). Different regulomeDB score (1–6) is represent the different degree for functional variant as transcription factor binding site. Score 1 indicates the strongest evidence for a SNP being located in a functional region.

Phyre2 (http://www.sbg.bio.ic.ac.uk/~phyre2/html/page.cgi?id=index) is an online tool to predict and analyze the secondary and tertiary structure and function of proteins[36]. Users can submit a number of sequences of amino acid, Phyre2 will predict the secondary and tertiary structure of their models automatically.

## Results

We found 128 OP-associated SNPs (S1 Table) and 157 associated genes by searching the PheGenI. We detected 88 genes for the 128 SNPs by GRAIL analysis: 31 genes can also be detected by PheGenI and 57 newly detected genes were. As shown in S1 Table, the columns “Gene 1” and “Gene 2” listed 157 unique genes, which were physically located at two sides of the corresponding 128 SNPs, and the “implicated genes” listed 88 unique genes from GRAIL analysis.

eQTL analysis[37–39] is an effective method to detect the functional mechanism underlying association by detecting the association between the genetic variants at DNA level and the variations in mRNA expression of genes near SNPs. Among the 128 unique SNPs, we found that 8 SNPs have potential eQTL effects on a total of eleven eQTL target genes (Table 1) in LCLS or monocytes[28–30] which are two type of cells closely related with bone metabolism and all of them act as cis-effect regulators.

**Table 1:** Expression quantitative trait locus (eQTL) analysis results between SNPs and the corresponding eQTL target genes.

SNP	Chr	position	Context	Allele	Association *P*-Value^1^	Gene1^2^	Gene2^2^	Implicated Gene^3^	eQTL Gene^4^	Effect^4^	Score^4^	Target^4^	Regulome DB score^6^
rs2278729	2	101668856	intron	A/G	1.00E-07	TBC1D8	TBC1D8	RPL31	RPL31	exon-QTL^5^	7.87	monocytes	1f
rs13182402	5	125918148	intron	A/G	2.00E-09	ALDH7A1	ALDH7A1	ALDH7A1	ALDH7A1	eQTL	45.14	LCLS	5
rs227584	17	42225547	missense	G/T	9.00E-07	C17orf53	C17orf53	SLC4A1	C17orf53	eQTL	35.55	LCLS	6
									TMUB2	eQTL	55.47	LCLS	
rs228769	17	42193185	intron	G/C	2.00E-08	HDAC5	HDAC5	SLC4A1	C17orf65	eQTL	32.60	monocytes	4
rs1007738	11	46849360	intron	G/A	7.00E-07	CKAP5	CKAP5	CREB3L1	MTCH2	exon-QTL	3.59	LCLS	1f
									PHF21A	exon-QTL	3.91	LCLS	
rs3736228	11	68201295	missense	C/T	6.00E-12	LRP5	LRP5	LRP5	CPT1A	eQTL	20.78	monocytes	1f
									MTL5	eQTL	13.26	monocytes	
rs1038304	6	151933175	intron	A/G	4.00E-11	C6orf97	C6orf97	C6orf97	C6orf97	eQTL	12.56	monocytes	6
rs884205	18	60054857	intergenic	T/G	9.00E-09	TNFRSF11A	RPL17P44	TNFRSF11A	TNFRSF11A	eQTL	1.00	monocytes	5

Note

^1^“Association *P*-value” are the results of PheGenI.

^2^“Gene 1” and “Gene 2” are the nearby genes physically located at two sides of the SNP.

^3^“Implicated genes" are the results from Gene Relationships Across Implicated Loci (GRAIL) analysis.

^4^"eQTL gene", "Effect", "Score", "Target" are the result of eQTL analysis. Score = −log_10_(*P*-value). The *P*-values represented the differences of gene expression of the different genotypes.

^5^“exon-QTL”: quantified reads for known exons by RNA sequencing against SNPs. All the eQTL genes have cis-effect.

^6^"RegulomeDB Score" is a scoring scheme of RegulomeDB and represents the source of supporting evidence. Among those, "1f" refers to the evidence from "eQTL + TF binding/DNase peak","4" refers to TF binding + DNase peak, "5" refers to TF binding or DNase peak, "6" refers to the evidence from "other"LCLs: lymphoblastoid cell lines.

Then, the differential expression analyses for the 11 identified eQTL target genes from the above 8 SNPs with potential eQTL effects in monocytes and B Lymphocytes was performed (Table 2). B cells are known regulators of bone metabolism, particularly for osteoclastogenesis[40,41]. We used the *t*-tests to compare the mRNA expression levels of the 11 eQTL target genes between high-BMD subjects and low-BMD subjects. The gene ribosomal protein L31 (*RPL31)* showed significantly differential expressions in GSE7158 and GSE13850 cell samples, with p value<0.05(GSE7158: *P* = 4.73E-02, GSE13850: *P* = 5.42E-03). The genes carnitine palmitoyltransferase 1A (*CPT1A)* and metallothionein-like 5 (*MTL5)* showed significantly differential expressions in GSE13850 cell sample(*CPT1A*:*P* = 1.56E-02, *MTL5*: *P* = 4.83E-02) (Table 2). The three genes were upregulated in the high bone mass group.

**Table 2:** Differential expression analysis for eQTL target genes in OP-related cells groups.

Sample	S1		S2		S3	
**GSE NO.**^1^	GSE7158		GSE2208		GSE13850	
**Disease**	osteoporosis		osteoporosis		osteoporosis	
**Target Cell**	Circulating Monocytes		Circulating Monocytes		Circulating B Lymphocytes	
**Sample size**^2^	12:14		9:10		10:10	
**Platform**	Affymetrix Human Genome U133 Plus 2.0		Affymetrix Human Genome U133A Array		Affymetrix Human Genome U133A Array	
**Gene Sample**	**Probe ID**^4^**(S1)**	*P***-value**	**Probe ID**^4^**(S2)**	*P***-value**	**Probe ID**^4^**(S3)**	*P***-value**
*RPL31*	241017_at	4.73E-02	200962_at	1.18E-01	200962_at	5.42E-03
*CPT1A*	203633_at	1.31E-01	203633_at	4.55E-01	210688_s_at	1.56E-02
*MTL5*	238246_at	1.21E-01	N/A^3^	N/A^3^	219786_at	4.83E-02

Note

^1^GSE NO: Gene Expression Omnibus Number (GSE7158[26], GSE2208[27], GSE13850[28]).

^2^Sample size: Low BMD:High BMD.

^3^N/A: Not available.

^4^We only listed the most significant expression results of probes if one gene has multiple detected probes.

We used the Regulome DB to investigate whether the 8 eQTL SNPs are functional variants with putative regulation effects. As shown in Table 1, three SNPs (*RPL31* rs2278729, *CREB3L1* rs1007738 and *LRP5* rs3736228), scored 1f, were annotated as the most credible functional variants with potential regulation effects and likely accounted for allelic-specific expressions of the 5 eQTL target genes (*RPL31*, *CPT1A*, *MTL5*, *MTCH2*, *PHF21A*).

**Fig 1:**
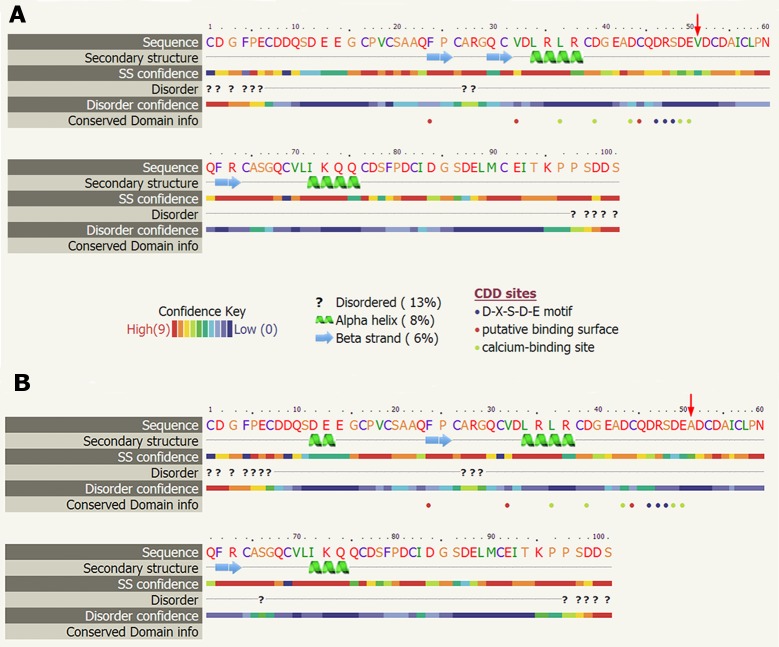
**Predicted secondary structure of protein carrying either (A) rs3736228-C allele or (B) rs3736228-T allele.** A:before missense mutation; B: after missense mutation. NOTE: The arrows point the position of the missense mutation. The 50 amino acids before and after missense mutations, which are: cdgfpecddqsdeegcpvcsaaqfpcargqcvdlrlrcdgeadcqdrsdeadcdaiclpnqfrcasgqcvlikqqcdsfpdcidgsdelmceitkppsdds and cdgfpecddqsdeegcpvcsaaqfpcargqcvdlrlrcdgeadcqdrsdevdcdaiclpnqfrcasgqcvlikqqcdsfpdcidgsdelmceitkppsdds, respectively.

Using the F-SNP program, we found that SNP rs3736228 located in nonsynonymous region and alteration of allele C to T caused a missense mutation of *LRP5* protein with amino acid residue alteration from Alanine (A) to Valine (V).We used 101 amino acids (50 amino acids before and 50 after the missense mutation of rs3736228) to predict the secondary structure of protein. We found that the amino acid residue alteration from Alanine (A) to Valine (V) caused loss of alpha helix and gain of beta strand before the missense mutation (Fig 1). We speculate that this SNP may influence osteoporosis risk through changing the amino acid sequence and secondary structure of the LRP5 protein.

## Discussion

In this study, we performed integrative analyses to explore functional mechanisms underlying the associations for OP by using publically available datasets. A total of 8 SNPs (rs2278729, rs13182402, rs227584, rs228769, rs1007738, rs3736228, rs1038304, rs884205) acting as cis-effect regulators on the 11 corresponding eQTL genes (*RPL31*, *ALDH7A1*, *C17orf53*, *TMUB2*, *C17orf65*,*MTCH2*, *PHF21A*, *CPT1A*, *MTL5*, *C6orf97*,*ENSG0000014*) were identified. Among these SNPs, two eQTL SNPs (*RPL31* rs2278729 and *LRP5* rs3736228) likely accounted for allelic-specific expressions of the 3 eQTL target genes (*CPT1A*, *MTL5* and *RPL31*) which showed significantly differential expression in monocytes and LCLs.

Integrative analyses by utilizing the public data resources can provide new understandings into the molecular genetic mechanisms of human diseases. In genetics, the genetic information carried on DNA is transferred to RNA through transcription and then translated into protein molecules[5]. The functional mechanisms for OP-associated genetic variants at the DNA level may be that genetic variants lead to variation of gene expression, and then cause the variation of susceptibility to OP[5]. Thus, integrating abundant evidences from multiple levels can help us to understand potential functional mechanism of genes and their contribution to variation in susceptibility to OP. We draw out the robust genetic associations between 2 SNPs of the corresponding genes (*CPT1A*, *MTL5*, *CREB3L1* and *RPL31*) and OP.

The previous study showed that rs3736228 in the *LRP5* gene was strongly associated with the BMD[42]. Through the eQTL analysis, the SNP rs3736228 may serve as a cis-effect regulator of genes *CPT1A* and *MTL5*[19]. *CPT1A* is a key regulator to facilitate the transfer of long-chain fatty acids across the mitochondrial membrane for β-oxidation in mammals[43]. *MTL5* may play a central role in the regulation of cell growth and differentiation[44]. Previous studies showed *CPT1A* and *MTL*5[45]genes were associated with lumbar spine BMD and *CPT1A* located in BIOCARTA_LEPTIN_PATHWAY which is important in the biology and etiology of osteoporosis[12]. In this study, we found the gene *CPT1A* and *MTL5* in B cells were significantly differential expressed between high and low BMD group (*P*<0.05). SNP rs3736228 acts as potential eQTL cis-effect on *CPT1A* and and *MTL5*. Therefore, we speculate that rs3736228 plays important roles in the pathological mechanism of osteoporosis by regulating differential mRNA transcriptions of *CPT1A* and *MTL5*. On the other hand, since rs3736228 caused a missense mutation for LRP5 protein, *LRP5* rs3736228 may influence and even induce osteoporosis through changing the amino acid sequence and secondary structure of *LRP5* protein. Our finding strongly suggested that rs3736228 was a causal variant functioning either by regulating expression of *CPT1A* and *MTL5* and/or by influencing function of LRP5 protein. Further functional study on the molecular mechanism of *CPT1A*, *MTL5* gene and *LRP5* rs3736228 may help us comprehend the association between osteoporosis and candidate gene, understand the pathogenesis of osteoporosis and the genetic mechanism.

*RPL31*, belongs to the L31E family of ribosomal proteins. The encoded protein is one of the members of the ribosomal 60S subunit. Ribosomal proteins have a lot of functions, such as participation in regulation of gene transcription and translation, involved in DNA repair, regulation of cell proliferation, differentiation, apoptosis and so on. In addition, ribosomal proteins may play an important role in tumor occurrence, development, metastasis and tumor suppression[46,47]. Allele A of rs2278729 was previously reported to be associated with smaller femoral neck-shaft angle in men and lower *RPL31* expression in lymphoc-ytes and osteoblasts. In this integrative study, we confirmed that SNP rs2278729 acts as cis-effect regulators for *RPL31*. We speculate that rs2278729 can regulate differential expression of protein by regulating differential mRNA transcriptions of gene *RPL31*, therefore, rs2278729 plays important roles in the variation of susceptibility to OP.

We identified two SNPs having combined OP-associated functional evidence among the searched 128 OP-associated SNPs. Failure to find functional evidence for some promising functional SNPs in BMD hot loci, like RANKL-OPG, ESR and LRP5 in this study does not exclude the importance of the associations of the BMD hot loci with OP. Instead, those associated SNPs may be involved in pathogenesis of OP by other mechanisms (such as epigenetic regulation or protein translation) instead of directly regulating target gene expression. Small effect size of the SNPs and small sample size will lead to limited statistical power, which is a reason for the lack of association between OP-associated SNPs and mRNA expression. Further functional studies are required to understand the functional association between genetic variants and OP, which may provide new insights into mechanisms underlying the association detected at DNA level.

There are two limitations to this study. First, although we identified some functional evidence for the associations between 2 SNPs and OP, it is still needed to further study the causal variants with potential functional effects. Second, although we used two methods to select the candidate genes, there is still another possibilities that other genes near the associated-SNPs are also directly or indirectly involved in the association around the SNPs[5]. In short, this is not a perfect method by public data sources to research the functional mechanisms of diseases, but to some degree, this is a simple, complementary, and effective way to reveal the functional link underlying the association between SNPs and diseases.

In summary, all of the functional analysis results we found in this study demonstrated the significance of the 2 candidate OP-associated SNPs (rs2278729 and rs3736228). We confirmed that the 2 SNPs have cis-regulation effects on the expression of 3 genes (*RPL31*,*CPT1A and MTL5*), at the same time, these 3 genes were differentially expressed between high BMD group and low BMD group. The above evidence provides some important clues for us to perform further study on the functional molecular mechanism between OP and OP-associated genetic variants. Performing the integrative analyses by utilizing the public data resources may provide a new perspective into the functional molecular genetic mechanisms underlying human complex diseases.

## Supporting information

S1 Table Information for the 128 genetic associations. N/A: not available. “Associated P-value”, “Source”, "PMID", “Gene 1” and “Gene 2” are the results of Phenotype-Genotype Integrator (PheGenI) and the “Gene 1” and “Gene 2” represent the nearest genes physically located at two sides of the SNP. "Implicated gene" are the results from Gene Relationships Across Implicated Loci (GRAIL) analysis. PMID: Pubmed ID for association results."RegulomeDB Score" is a scoring scheme of RegulomeDB and represents the source of supporting evidence. Among those, "1f" refers to the evidence from "eQTL + TF binding/DNase peak", “2b” refers to TF binding + any motif + DNase Footprint + DNase peak”, “3a” refers to TF binding + any motif + DNase peak, "4" refers to TF binding + DNase peak, "5" refers to TF binding or DNase peak, "6" refers to the evidence from "other"LCLs: lymphoblastoid cell lines.
(DOCX)
