# Supplementary material for: Functional relevance for associations between osteoporosis and genetic variants
Source: PLoS One. 2017 Apr 3;12(4):e0174808. doi: 10.1371/journal.pone.0174808 (PMC5378394; doi:10.1371/journal.pone.0174808)
Supplement: S1 Table — N/A: not available. “Associated P-value”, “Source”, "PMID", “Gene 1” and “Gene 2” are the results of Phenotype-Genotype Integrator (PheGenI) and the “Gene 1” and “Gene 2” represent the nearest genes physically located at two sides of the SNP. "Implicated gene" are the results from Gene Relationships Across Implicated Loci (GRAIL) analysis. PMID: Pubmed ID for association results."RegulomeDB Score" is a scoring scheme of RegulomeDB and represents the source of supporting evidence. Among those, "1f" refers to the evidence from "eQTL + TF binding/DNase peak", “2b” refers to TF binding + any motif + DNase Footprint + DNase peak”, “3a” refers to TF binding + any motif + DNase peak, "4" refers to TF binding + DNase peak, "5" refers to TF binding or DNase peak, "6" refers to the evidence from "other"LCLs: lymphoblastoid cell lines. (DOCX) [file pone.0174808.s001.docx]

**Supporting Information**

**S1 Table. Information for the 128 genetic associations**

| **SNP** | **Chr** | **Location** | **Context** | **Gene1** | **Gene2** | **Association P-Value** | **Implicated Gene** | **PMID** | **Regulome DB score** |
| --- | --- | --- | --- | --- | --- | --- | --- | --- | --- |
|  |  |  |  |  |  |  |  |  |  |
| rs87938 | 3 | 41137672 | intergenic | RPS27P4 | MRPS31P1 | 8.00E-10 | CTNNB1 | 19801982 | 6 |
| rs199055 | 6 | 23490117 | intergenic | RPL6P18 | NRSN1 | 5.36E-08 | N/A | 17903296 | No Data |
| rs227584 | 17 | 42225547 | missense | C17orf53 | C17orf53 | 9.00E-07 | SLC4A1 | 19079262 | 6 |
| rs228769 | 17 | 42193185 | intron | HDAC5 | HDAC5 | 2.00E-08 | SLC4A1 | 19801982 | 4 |
| rs340707 | 8 | 139379964 | intron | FAM135B | FAM135B | 4.32E-10 | FAM135B | 17903295 | 6 |
| rs344720 | 5 | 16205201 | intergenic | ZNF622 | ZNF622 | 5.07E-07 | ZNF622 | 17903296 | 6 |
| rs494453 | 1 | 112192122 | intergenic | ADORA3 | RAP1A | 4.00E-08 | RAP1A | 20548944 | 5 |
| rs512189 | 1 | 182632587 | intron | RGS8 | RGS8 | 4.40E-07 | N/A | 17903296 | No Data |
| rs585966 | 1 | 182632187 | intron | RGS8 | RGS8 | 5.20E-07 | N/A | 17903296 | 6 |
| rs599083 | 11 | 68192346 | intron | LRP5 | LRP5 | 5.00E-08 | LRP5 | 19801982 | 5 |
| rs719988 | X | 85148366 | intron | CHM | CHM | 1.73E-07 | CHM | 17903295 | 6 |
| rs884205 | 18 | 60054857 | intergenic | TNFRSF11A | RPL17P44 | 9.00E-09 | TNFRSF11A | 19801982 | 5 |
| rs947425 | 1 | 119863356 | intergenic | RPL6P2 | HAO2 | 5.89E-07 | N/A | 17903296 | 5 |
| rs1007738 | 11 | 46849360 | intron | CKAP5 | CKAP5 | 7.00E-07 | CREB3L1 | 19079262 | 1f |
| rs1021188 | 13 | 43116133 | intergenic | FABP3P2 | TNFSF11 | 2.00E-14 | TNFSF11 | 21124946 | 6 |
| rs1038304 | 6 | 151933175 | intron | C6orf97 | C6orf97 | 4.00E-11 | C6orf97 | 18445777 | 6 |
| rs1054627 | 4 | 88732692 | missense | IBSP | IBSP | 8.00E-07 | IBSP | 21533022 | 5 |
| rs1366594 | 5 | 88376061 | intergenic | MEF2C | CETN3 | 1.00E-13 | MEF2C | 19801982 | No Data |
| rs1407015 | 20 | 31809852 | intron | C20orf71 | C20orf71 | 1.91E-13 | N/A | 17903296 | 6 |
| rs1430742 | 1 | 68635075 | intron | WLS | WLS | 3.00E-13 | GPR177 | 19801982 | 6 |
| rs1438029 | 2 | 140451208 | intergenic | MRPS18BP2 | RPL9P13 | 4.57E-08 | LRP1B | 17903296 | 6 |
| rs1463605 | 12 | 30113883 | intergenic | RPL21P99 | IPO8 | 6.96E-08 | TMTC1 | 17903295 | No Data |
| rs1471403 | 4 | 88775243 | intergenic | MEPE | HSP90AB3P | 2.00E-08 | MEPE | 19801982 | 5 |
| rs1498744 | 12 | 77556602 | intergenic | E2F7 | NAV3 | 4.35E-07 | E2F7 | 17903296 | No Data |
| rs1513670 | 17 | 41807331 | intergenic | WHSC1L2P | SOST | 2.00E-08 | SOST | 19079262 | No Data |
| rs1524058 | 7 | 38136277 | intergenic | EPDR1 | STARD3NL | 1.00E-09 | SFRP4 | 19801982 | No Data |
| rs1561164 | 3 | 87126026 | intergenic | VGLL3 | CHMP2B | 1.87E-08 | POU1F1 | 17903295 | No Data |
| rs1647978 | 15 | 60939249 | intron | RORA | RORA | 1.11E-08 | RORA | 17903296 | 5 |
| rs1805880 | 8 | 91032188 | intron | DECR1 | DECR1 | 2.00E-09 | CALB1 | 17903296 | No Data |
| rs1858114 | 4 | 138735718 | intergenic | PCDH18 | SLC7A11 | 1.48E-11 | SLC7A11 | 17903295 | 6 |
| rs1926289 | 1 | 68465932 | intergenic | RNU7-80P | DIRAS3 | 4.89E-07 | DIRAS3 | 17903296 | No Data |
| rs1943212 | 18 | 73442451 | intergenic | C18orf62 | ZNF516 | 1.12E-09 | C18orf62 | 17903295 | 6 |
| rs1999805 | 6 | 152068364 | intron | ESR1 | ESR1 | 2.00E-08 | ESR1 | 18445777 | 5 |
| rs2016266 | 12 | 53727955 | intron | SP7 | SP7 | 1.00E-08 | SP7 | 19801982 | 5 |
| rs2062375 | 8 | 119977792 | intergenic | TNFRSF11B | COLEC10 | 3.00E-11 | TNFRSF11B | 20548944 | 6 |
| rs2062377 | 8 | 120007420 | intergenic | TNFRSF11B | COLEC10 | 4.00E-16 | TNFRSF11B | 19801982 | 5 |
| rs2096437 | 1 | 45748381 | intergenic | ZSWIM5 | HPDL | 1.65E-07 | N/A | 17903296 | 6 |
| rs2101552 | 14 | 63192948 | intron | KCNH5 | KCNH5 | 6.11E-07 | KCNH5 | 17903295 | No Data |
| rs2164531 | 4 | 148850967 | intron | ARHGAP10 | ARHGAP10 | 1.14E-12 | ARHGAP10 | 17903296 | 6 |
| rs2165468 | 10 | 3516105 | intergenic | PITRM1 | KLF6 | 1.00E-06 | KLF6 | 17903296 | No Data |
| rs2241023 | 2 | 160081336 | intron | TANC1 | TANC1 | 2.01E-07 | WDSUB1 | 17903296 | 6 |
| rs2273061 | 20 | 10639543 | intron | JAG1 | JAG1 | 5.00E-08 | JAG1 | 20096396 | 2b |
| rs2278729 | 2 | 101668857 | intron | TBC1D8 | TBC1D8 | 1.00E-07 | RPL31 | 20548944 | 1f |
| rs2336434 | 12 | 65519266 | intergenic | WIF1 | LEMD3 | 1.91E-07 | WIF1 | 17903296 | 4 |
| rs2424513 | 20 | 23046024 | intergenic | THBD | CD93 | 3.33E-13 | CD93 | 17903295 | 5 |
| rs2504063 | 6 | 152090707 | intron | ESR1 | ESR1 | 6.00E-11 | ESR1 | 19801982 | No Data |
| rs2566755 | 1 | 68635390 | intron | WLS | WLS | 2.00E-12 | GPR177 | 19801982 | 5 |
| rs2710057 | X | 86192003 | intergenic | BA345E19.2 | KLHL4 | 1.00E-06 | DACH2 | 19079262 | No Data |
| rs2886792 | 7 | 80376261 | intron | SEMA3C | SEMA3C | 1.75E-07 | SEMA3C | 17903296 | 6 |
| rs2941740 | 6 | 152009638 | nearGene-5 | ESR1 | ESR1 | 2.00E-10 | ESR1 | 19801982 | 5 |
| rs3018362 | 18 | 60082093 | intergenic | TNFRSF11A | RPL17P44 | 1.00E-06 | TNFRSF11A | 18445777 | 4 |
| rs3130340 | 6 | 32244627 | intergenic | NOTCH4 | C6orf10 | 1.00E-07 | BTNL2 | 18445777 | No Data |
| rs3736228 | 11 | 68201295 | missense | LRP5 | LRP5 | 6.00E-12 | LRP5 | 18455228 | 1f |
| rs3762397 | 1 | 200090219 | intron | NR5A2 | NR5A2 | 2.78E-07 | NR5A2 | 17903296 | No Data |
| rs4087296 | 16 | 82377781 | intergenic | MPHOSPH6 | CDH13 | 3.11E-07 | CDH13 | 17903296 | 3a |
| rs4338582 | 12 | 43007527 | intergenic | PRICKLE1 | RPS27P21 | 1.69E-09 | N/A | 17903296 | 5 |
| rs4355801 | 8 | 119923873 | intergenic | RPS26P35 | TNFRSF11B | 8.00E-10 | TNFRSF11B | 18455228 | No Data |
| rs4604027 | 4 | 72133586 | intron | SLC4A4 | SLC4A4 | 4.06E-08 | SLC4A4 | 17903296 | No Data |
| rs4670136 | 2 | 36305239 | intergenic | MRPL50P1 | RPL21P36 | 1.70E-13 | CRIM1 | 17903295 | 6 |
| rs4729260 | 7 | 96117918 | intron | FLJ42280 | FLJ42280 | 2.00E-10 | SLC25A13 | 19801982 | 6 |
| rs4811196 | 20 | 36469694 | intron | CTNNBL1 | CTNNBL1 | 1.00E-06 | CTNNBL1 | 17903296 | 6 |
| rs4870044 | 6 | 151901409 | intron | C6orf97 | C6orf97 | 2.00E-11 | C6orf97 | 18445777 | 5 |
| rs5917609 | X | 38475385 | intron | TSPAN7 | TSPAN7 | 9.40E-08 | TSPAN7 | 17903296 | No Data |
| rs6426749 | 1 | 22711473 | intergenic | WNT4 | ZBTB40 | 9.00E-08 | WNT4 | 19801982 | 6 |
| rs6469804 | 8 | 120044829 | intergenic | TNFRSF11B | COLEC10 | 7.00E-15 | TNFRSF11B | 18445777 | 6 |
| rs6632753 | X | 13368071 | intergenic | ATXN3L | EGFL6 | 2.79E-08 | RAB9A | 17903296 | 5 |
| rs6696981 | 1 | 22702858 | intergenic | WNT4 | ZBTB40 | 2.00E-08 | WNT4 | 19079262 | 5 |
| rs6710518 | 2 | 166583244 | intergenic | CSRNP3 | GALNT3 | 5.00E-10 | GALNT3 | 21533022 | No Data |
| rs6929137 | 6 | 151936677 | missense | C6orf97 | C6orf97 | 2.00E-10 | C6orf97 | 19079262 | 5 |
| rs6993813 | 8 | 120052238 | intergenic | TNFRSF11B | COLEC10 | 3.00E-11 | TNFRSF11B | 18445777 | No Data |
| rs7089474 | 10 | 133509704 | intergenic | C10orf90 | DOCK1 | 1.14E-17 | N/A | 17903296 | No Data |
| rs7112939 | 11 | 80033411 | intergenic | ODZ4 | RPS28P7 | 5.70E-12 | N/A | 17903296 | No Data |
| rs7113470 | 11 | 80033803 | intergenic | ODZ4 | RPS28P7 | 5.70E-12 | N/A | 17903296 | 3a |
| rs7117858 | 11 | 15694462 | intergenic | INSC | SOX6 | 6.00E-10 | SOX6 | 19801982 | 5 |
| rs7227401 | 18 | 21938658 | intron | OSBPL1A | OSBPL1A | 4.00E-07 | OSBPL1A | 20548944 | No Data |
| rs7524102 | 1 | 22698447 | intergenic | WNT4 | ZBTB40 | 1.00E-16 | WNT4 | 19079262 | 5 |
| rs7605378 | 2 | 200676926 | intergenic | FLJ32063 | C2orf69 | 2.00E-08 | LOC26010 | 21573133 | 6 |
| rs7776725 | 7 | 121033121 | intron | FAM3C | FAM3C | 1.00E-11 | WNT16 | 19396169 | 5 |
| rs7781370 | 7 | 96133531 | intergenic | FLJ42280 | SHFM1 | 5.00E-12 | SLC25A13 | 19801982 | 6 |
| rs7888911 | X | 32561313 | intron | DMD | DMD | 6.06E-08 | DMD | 17903295 | 6 |
| rs7932354 | 11 | 46722221 | nearGene-5 | ZNF408 | ZNF408 | 4.00E-09 | CREB3L1 | 19801982 | 4 |
| rs8095315 | 18 | 73443682 | intergenic | C18orf62 | ZNF516 | 3.19E-08 | C18orf62 | 17903295 | 5 |
| rs9287785 | 2 | 160075009 | intron | TANC1 | TANC1 | 3.97E-09 | WDSUB1 | 17903296 | 5 |
| rs9303521 | 17 | 43805194 | intergenic | C17orf69 | CRHR1 | 1.00E-08 | MAPT | 19801982 | 6 |
| rs9317284 | 13 | 63634350 | intergenic | RPL32P28 | OR7E156P | 2.00E-07 | N/A | 17903296 | 5 |
| rs9533090 | 13 | 42951449 | intergenic | FABP3P2 | TNFSF11 | 5.00E-25 | TNFSF11 | 19801982 | No Data |
| rs9594738 | 13 | 42952145 | intergenic | FABP3P2 | TNFSF11 | 2.00E-08 | TNFSF11 | 18445777 | 4 |
| rs9594759 | 13 | 43032593 | intergenic | FABP3P2 | TNFSF11 | 2.00E-21 | TNFSF11 | 18445777 | No Data |
| rs9630182 | 11 | 13620172 | intergenic | PTH | FAR1 | 4.00E-07 | PTH | 19874204 | 6 |
| rs10048146 | 16 | 86710660 | intergenic | FOXL1 | RPL39P30 | 2.00E-08 | FOXL1 | 19801982 | 5 |
| rs10484990 | 6 | 54548309 | intergenic | RPSAP44 | KRASP1 | 3.43E-08 | FAM83B | 17903295 | No Data |
| rs10486031 | 7 | 14485463 | intron | DGKB | DGKB | 1.65E-07 | DGKB | 17903296 | 6 |
| rs10488771 | 11 | 73994285 | intron | P4HA3 | P4HA3 | 9.37E-07 | PPME1 | 17903296 | 3a |
| rs10490003 | 2 | 160082890 | intron | TANC1 | TANC1 | 6.50E-07 | WDSUB1 | 17903296 | 3a |
| rs10490004 | 2 | 160076140 | intron | TANC1 | TANC1 | 2.34E-08 | WDSUB1 | 17903296 | 6 |
| rs10490005 | 2 | 160075645 | intron | TANC1 | TANC1 | 9.55E-08 | WDSUB1 | 17903296 | No Data |
| rs10490481 | 2 | 15220044 | intergenic | FAM84A | NBAS | 2.48E-07 | NAG | 17903296 | 5 |
| rs10490661 | 2 | 36967768 | intron | VIT | VIT | 4.97E-10 | VIT | 17903296 | 6 |
| rs10491303 | 5 | 136226548 | intergenic | TRPC7 | SPOCK1 | 3.17E-09 | N/A | 17903296 | 6 |
| rs10492713 | 13 | 108177428 | intron | FAM155A | FAM155A | 1.26E-08 | LOC728215 | 17903296 | No Data |
| rs10493509 | 1 | 73209817 | intergenic | RPL31P12 | KRT8P21 | 2.64E-07 | N/A | 17903295 | No Data |
| rs10494675 | 1 | 193150873 | intron | CDC73 | CDC73 | 3.51E-09 | CDC73 | 17903295 | 5 |
| rs10495089 | 1 | 218126346 | intergenic | UBBP2 | RRP15 | 3.27E-08 | TGFB2 | 17903296 | 5 |
| rs10497907 | 2 | 209490753 | intergenic | PTH2R | CRYGFP | 7.88E-07 | N/A | 17903296 | 6 |
| rs10501098 | 11 | 28436499 | intergenic | RPS15AP31 | OR2BH1P | 4.21E-08 | KIF18A | 17903296 | No Data |
| rs10506532 | 12 | 65505662 | intron | WIF1 | WIF1 | 1.85E-07 | WIF1 | 17903296 | 5 |
| rs10506701 | 12 | 74586210 | intergenic | RPL31P48 | VENTXP3 | 1.00E-06 | N/A | 17903296 | No Data |
| rs10506821 | 12 | 80496923 | intergenic | RPL26P32 | RPL26P32 | 1.42E-07 | PPP1R12A | 17903295 | 6 |
| rs10511321 | 3 | 112913505 | intergenic | C3orf17 | BOC | 3.98E-07 | N/A | 17903296 | 5 |
| rs10514102 | 18 | 71614819 | intergenic | NETO1 | FBXO15 | 8.71E-16 | FBXO15 | 17903296 | 3a |
| rs10516293 | 4 | 15850110 | intron | CD38 | CD38 | 3.38E-07 | N/A | 17903296 | 4 |
| rs10518632 | 4 | 134167966 | intergenic | PCDH10 | PABPC4L | 3.65E-07 | PCDH10 | 17903296 | No Data |
| rs10518712 | 15 | 41372239 | intron | INO80 | INO80 | 4.14E-07 | N/A | 17903296 | 2 |
| rs10520144 | 15 | 39943643 | intron | FSIP1 | FSIP1 | 1.13E-09 | THBS1 | 17903296 | No Data |
| rs10520420 | 4 | 180139179 | intergenic | RPL19P8 | MGC45800 | 4.07E-07 | N/A | 17903296 | No Data |
| rs10876432 | 12 | 53731891 | intergenic | SP7 | SP1 | 1.00E-07 | SP7 | 19079262 | 4 |
| rs11023787 | 11 | 15952294 | intergenic | INSC | SOX6 | 5.00E-16 | SOX6 | 21104366 | 5 |
| rs11864477 | 16 | 77427769 | intron | ADAMTS18 | ADAMTS18 | 2.00E-08 | ADAMTS18 | 19249006 | 5 |
| rs11898505 | 2 | 54684557 | intron | SPTBN1 | SPTBN1 | 8.00E-07 | SPTBN1 | 18445777 | 2b |
| rs11995824 | 8 | 120012700 | intergenic | TNFRSF11B | COLEC10 | 7.00E-09 | TNFRSF11B | 19801982 | 5 |
| rs12151790 | 2 | 235210727 | intergenic | SPP2 | RPS20P12 | 5.00E-07 | ARL4C | 20548944 | 5 |
| rs13308199 | 11 | 39327959 | intergenic | RPL18P8 | LRRC4C | 9.00E-07 | N/A | 20548944 | No Data |
| rs13182402 | 5 | 125918148 | intron | ALDH7A1 | ALDH7A1 | 2.00E-09 | ALDH7A1 | 20072603 | 5 |
| rs13204965 | 6 | 127167072 | intergenic | RPS4XP9 | RSPO3 | 3.00E-08 | C6orf173 | 21533022 | 6 |
| rs16921914 | 11 | 31210771 | intergenic | DCDC5 | DCDC1 | 2.00E-09 | DCDC1 | 19801982 | No Data |
| rs16963644 | 15 | 50638677 | intron | GABPB1 | GABPB1 | 3.10E-11 | GABPB2 | 17903296 | 5 |
| rs17131547 | 1 | 92211020 | intron | TGFBR3 | TGFBR3 | 1.00E-06 | N/A | 19249006 | 4 |
| rs17184557 | 18 | 67142857 | Intron | DOK6 | DOK6 | 9.00E-07 | DOK6 | 20548944 | 6 |

NOTE:

N/A: not available.
“Associated P-value”, “Source”, "PMID", “Gene 1” and “Gene 2” are the results of Phenotype-Genotype Integrator (PheGenI) and the “Gene 1” and “Gene 2” represent the nearest genes physically located at two sides of the SNP.

"Implicated gene" are the results from Gene Relationships Across Implicated Loci (GRAIL) analysis.

PMID: Pubmed ID for association results.

"RegulomeDB Score" is a scoring scheme of RegulomeDB and represents the source of supporting evidence. Among those, "1f" refers to the evidence from "eQTL + TF binding/DNase peak", “2b” refers to TF binding + any motif + DNase Footprint + DNase peak”, “3a” refers to TF binding + any motif + DNase peak, "4" refers to TF binding + DNase peak, "5" refers to TF binding or DNase peak, "6" refers to the evidence from "other"LCLs: lymphoblastoid cell lines.
